# Risk factors for residual fibroglandular breast tissue following a mastectomy - an overview and retrospective cohort study

**DOI:** 10.1186/s12885-024-12491-4

**Published:** 2024-07-18

**Authors:** Deutschmann Christine, Singer F. Christian, Korbatits Ricarda, Kraus Christine, Gschwantler-Kaulich Daphne, Leser Carmen, Marzogi Alaa, Pascal A.T. Baltzer, Helbich H. Thomas, Pfeiler Georg, Clauser Paola

**Affiliations:** 1 Department of Obstetrics and Gynecology, Division of General Gynecology and Gynecologic Oncology, Medical University of Vienna, Waehringer Guertel 18-20, Vienna, 1090 Austria; 2 Department of Medical Imaging, King Abdullah Medical City Specialist Hospital, Muzdalifah Rd, Al Mashair, Makkah, 24246 Saudi Arabia; 3 Department of Biomedical Imaging and Image-guided Therapy, Division of General and Pediatric Radiology, Medical University of Vienna, Waehringer Guertel 18-20, Vienna, 1090 Austria

**Keywords:** Residual fibroglandular breast tissue, Mastectomy, Risk factor, Nipple-sparing mastectomy

## Abstract

**Background:**

Residual fibroglandular breast tissue (RFGT) following a mastectomy is associated with the remaining of occult breast cancer at the time of mastectomy as well as an increased local recurrence risk thereafter. Despite its oncologic implications, data on measures to prevent RFGT are lacking. Therefore, in a first step knowledge of risk factors for RFGT is of uttermost importance in order to allow identification of patients at risk and subsequently adaption of the surgical treatment and potentially prevention of RFGT a priori.

**Methods:**

We performed a systematic literature review in PubMed using the MESH terms [residual fibroglandular breast tissue], [residual breast tissue], [mastectomy] and [risk factor] followed by a retrospective data analysis including all patients with a mastectomy treated at the Department of Obstetrics and Gynecology of the Medical University of Vienna, Austria, between 01.01.2015 and 26.02.2020 in order to identify risk factors of RFGT following a mastectomy. The primary aim of the study was to assess a potential difference in RFGT volume between the different types of mastectomy. The secondary objectives of the study were to identify other potential risk factors for RFGT as well as to compare the skin and subcutaneous fat tissue thickness pre- to postoperatively.

**Results:**

Significantly higher RFGT volumes were observed following a nipple-sparing mastectomy (NSM) compared to a skin-sparing mastectomy (SSM) and radical mastectomy (RME) (*p* < .001). Furthermore, RFGT volume was significantly associated with the variables: reconstruction (*p* = .012), acellular dermal matrix (ADM) or mesh (*p* = .031), patient age (*p* = .022), preoperative fibroglandular tissue (FGT) volume (*p* = .012) and preoperative whole breast volume (including the skin envelope and nipple-areola-complex) (*p* = .030). The reduction in the postoperative compared to preoperative skin envelope thickness measured medially and laterally reached statistical significance in the NSM-cohort (medial *p* < .001, lateral *p* = .001) and showed a numerical difference in the RME and SSM-cohort.

**Conclusion:**

Mastectomy type, reconstruction, ADM or mesh, patient age, preoperative FGT volume and whole breast volume were identified as risk factors for RFGT in univariable analysis. The observed reduction in the post- compared to preoperative skin envelope thickness should be avoided considering the known associated increase in risk for ischemic complications.

**Supplementary Information:**

The online version contains supplementary material available at 10.1186/s12885-024-12491-4.

## Background

The aim of a mastectomy is the complete removal of fibroglandular breast tissue. Yet, due to the frequent irregularity or lack of the superficial fascia of the breast – which is considered as the landmark of dissection – fibroglandular breast tissue remains [1–18]. Furthermore, in some cases the subcutaneous tissue layer is simply too thin to allow total removal of breast tissue and simultaneously preservation of a well-perfused skin flap. Papassotiropoulos B et al. [12] demonstrated a distance of below 1 mm between the mastectomy specimen surface and breast tissue to be significantly associated with higher residual fibroglandular breast tissue (RFGT) rates in the skin flap.

RFGT has been observed in up to 100% of studied patients [19] and to amount up to 26% of the preoperative fibroglandular tissue (FGT) [3]. Unifocal [4] as well as multifocal dissemination [20] of RFGT has been described with various predilection sites including the craniolateral quadrant [3], the caudolateral quadrant [18] as well as the retroareolar region [19].

RFGT worsens the prognosis of the patient as it is associated with the remaining of occult breast cancer at the time of mastectomy as well as an increased local recurrence risk indefinitely after the surgery [7, 10, 16, 17, 21, 22].

In view of the oncologic consequences of RFGT and the lack of international consensus on the prevention of it – identification of patients at risk for RFGT in a first step is of uttermost clinical importance. Until now, only few authors have evaluated risk-factors for RFGT – and reported contradictory results [1–4, 8, 10–12, 14, 17–20, 23, 24]. Inconclusive findings were – among other parameters – described for type of mastectomy – which is in view of the rising nipple (NSM)- and skin-sparing mastectomy (SSM) rates particularly worth further studying.

Notably, besides the incomplete removal of FGT in the course of a mastectomy – the risk of a too radical surgical approach with additional resection of the subcutaneous tissue and subsequently increased risk of ischemic complications has been implied in literature and needs further evaluation [22].

## Aims

The primary aim of the study was to assess a potential difference in RFGT volume between the different types of mastectomies (radical mastectomy (RME) vs. SSM vs. NSM). The secondary objectives of the study were to identify other potential risk factors for RFGT following a mastectomy and to assess the postoperative skin envelope thickness in comparison to preoperative measurements.

## Methods

We performed a systematic literature search in PubMed using the MESH terms [residual fibroglandular breast tissue], [residual breast tissue], [mastectomy] and [risk factor] to provide an overview of risk factors for RFGT following a mastectomy.

We furthermore, performed a retrospective analysis of patients with a therapeutic or prophylactic mastectomy that were treated at the Department of Obstetrics and Gynecology of the Medical University of Vienna, Austria, between 01.01.2015 and 26.02.2020 and had an archived postoperative breast magnetic resonance imaging (MRI) examination. Patients with a second-look resection, an autologous breast reconstruction prior to the postoperative breast MRI and patients without an available post-mastectomy MRI or clinical data were excluded from the retrospective analysis.

Breast MRI examinations were done on 1.5T or 3T scanner following international guidelines [25], with dedicated coils and patients lying in a prone position. Measurements were conducted by an experienced breast radiologist. Pre-contrast T1-weighted 3D gradient-echo sequences and T2-weighted Turbo-Spin echo sequences were used to quantify RFGT. In addition, when available, preoperative MRI was used to aid in the correct identification of RFGT. MRI measurements included preoperative whole breast (including the skin envelope and nipple-areola-complex (NAC)), preoperative FGT, postoperative RFGT volume and the pre- and postoperative tissue envelope thickness.

Volumes were calculated with the help of a semi-automated segmentation with a dedicated software (ITK-SNAP) [26]. Measures are reported in mm^3^. RFGT thickness was determined in the retroareolar region and in the medial and lateral aspect of the breast. Measures are stated in mm.

Mean, standard deviation, minimum, maximum, median and interquartile range were used to describe metric variables. Frequencies and percentages were evaluated to describe nominal scaled parameters. The correlation between two nominal scaled variables was tested using Chi-square test – and if required - Fisher’s exact test. To test the difference between metric variables between 2 groups the students T-test and in case of skewed distribution of data Mann-Whitney-U-test were applied. In case of more than 2 groups the Kruskal-Wallis test was used.

Methods were previously presented in more detail in a study on the oncologic implications of RFGT based on the same study population [22].

The study was approved by the ethics´ committee of the Medical University of Vienna (Ethikkommission Medizinische Universitaet Wien, EK-Nr. 1067/2020). The need for informed consent was waived due to the retrospective nature of the study. The study was performed in accordance with the Declaration of Helsinki.

## Results

135 patients (181 breasts) of 737 screened patients (897 breasts) met the inclusion criteria and were enrolled in the study. An overview of excluded patients is presented in Supplement Materials (see Table S1).

A RME was performed on 81 breasts (44.8%), a NSM on 66 breasts (36.5%) and a SSM on 34 (18.8%) breasts. Median follow-up duration in the RME subgroup was 47.3 months (IQR 26.4; 82.4), 26.1 months (IQR 11.8; 44.2) in the SSM and 25.2 months (IQR 18.5; 38.0) in the NSM subgroup. Patient characteristics are displayed in Table 1. Details regarding the surgical procedure are presented in Table 2.

**Table 1:** Patient characteristics

	RME	SSM	NSM	*p*-VALUE
**No. patients/breasts (%)**	70/81 (44.8%)	25/34 (18.8%)	40/66 (36.5%)	
**Age** (median years, IQR)_PP_	52.4 (42.4; 59.3)	49.4 (41.7; 56.0)	38.0 (33.6; 48.3)	<0.001
**BMI** (median kg/m^2^, IQR)_PP_	25.6 (22.0; 28.9)	24.2 (21.3; 29.4)	22.8 (20.8; 25.7)	0.008
**Menopausal status**_PP_				<0.001
- premenopausal	27 (38.6%)	10 (41.7%) 1 missing value	32 (88.9%) 4 missing values	
- perimenopausal	2 (2.9%)	0 1 missing value	1 (2.8%) 4 missing values	
- postmenopausal	41 (58.6%)	14 (58.3%) 1 missing value	3 (8.3%) 4 missing values	
**Neoadjuvant chemotherapy (NACT)**_PB(only cases with oncologic mastectomy indication considered)_	22 (30.1%) 1 missing value	2 (9.5%)	16 (51.6%)	0.005
**Radiotherapy (RT)**_PB(only cases with oncologic mastectomy indication considered)_	24 (32.4%)	4 (19.0%)	2 (6.5%)	0.015
- prior to ME	1 (1.4%)	1 (4.8%)	0	
- post ME	23 (31.1%)	3 (14.3%)	2 (6.5%)	
**Prior breast operation**_PB_	18 (22.2%)	6 (17.6%)	9 (13.8%) 1 missing value	0.427
- Breast conserving surgery for BC	17	6	4	
- Breast conserving surgery for fibroadenoma, B3 lesion	0	0	2	
- Mastopexy or reduction mammaplasty	1	0	1	
- Breast augmentation with breast implants	0	0	2	

Patient characteristics, PP: analysis per patient, PB: analysis per breast, ME: mastectomy, BC: breast cancer, RME: radical mastectomy, SSM: skin-sparing mastectomy, NSM: nipple-sparing mastectomy

**Table 2:** Surgery

	RME	SSM	NSM	*p*-VALUE
**Mastectomy**				
**- Mastectomy indication**_PB (multiple answers per breast possible)_				
**- Oncologic**	74 (91.4%)	21 (61.8%)	31 (47.0%)	<0.001
- Primary BC	66 (81.5%)	20 (58.8%)	31 (47.0%)	
- In-breast local recurrence	8 (9.9%)	1 (2.9%)	0	
- New primary tumor	0	0	0	
**- Prophylactic**	12 (14.8%)	16 (47.1%)	49 (74.2%)	<0.001
- HBOC	8 (9.9%)	12 (35.3%)	40 (60.6%)	
- Mantle-field radiotherapy	0	0	2 (3.0%)	
- Undefined	4 (4.9%)	4 (11.8%)	7 (10.6%)	
**- ME volume** (mean g, IQR)_PB_	545.0 (540; 566)	510.0 (392; 666)	347.0 (204; 430)	0.001
**Lymph node surgery**_PB(only cases with oncologic mastectomy indication considered)_				0.015
- None	5 (7.0%) 3 missing values	2 (10.5%) 2 missing values	5 (16.1%)	
- Sentinel node	23 (32.4%) 3 missing values	11 (57.9%) 2 missing values	17 (54.8%)	
- Axillary dissection	43 (60.6%) 3 missing values	6 (31.6%) 2 missing values	9 (29.0%)	
**Reconstruction**				
**- Time of reconstruction and type of prothesis**_PB_				<0.001
- Immediate, primary-fixed volume implant	6 (27.3%)	23 (74.2%)	61 (98.4%)	
- Immediate, primary tissue expander	11 (50.0%)	8 (25.8%)	1 (1.6%)	
- Delayed, primary tissue expander	5 (22.7%)	0	0	
**- ADM and synthetic mesh**_PB_				<0.001
- ADM	7 (35.0%) 2 missing values	8 (29.6%) 4 missing values	8 (29.6%) 35 missing values	
- Synthetic mesh	1 (5.0%) 2 missing values	11 (40.7%) 4 missing values	11 (40.7%) 35 missing values	
- None	12 (60.0%) 2 missing values	8 (29.6%) 4 missing values	8 (29.6%) 35 missing values	

Surgery, PB: Analysis per breast, BC: breast cancer, RME: radical mastectomy, SSM: skin-sparing mastectomy, NSM: nipple-sparing mastectomy, ADM: acellular dermal matrix

91.4% (74/81) of RMEs were performed with an oncologic indication, compared to 61.8% (21/ 34) of SSMs and 47.0% (31/66) of NSMs. Details regarding breast cancer characteristics are displayed in Table 3.

**Table 3:** Breast cancer characteristics

	RME	SSM	NSM	*p*-VALUE
**Breast cancer characteristics at diagnosis**_PB(only cases with oncologic mastectomy indication considered)_				
- **BC subtype**_PB_				0.205
- Luminal A	21 (31.8%) 8 missing values	8 (40.0%) 1 missing value	6 (19.4%)	
- Luminal B	13 (19.7%) 8 missing values	4 (20.0%) 1 missing value	7 (22.6%)	
- Her2 enriched	24 (36.4%) 8 missing values	6 (30.0%) 1 missing value	8 (25.8%)	
- Triple negative	8 (12.1%) 8 missing values	2 (10.0%) 1 missing value	10 (32.3%)	
- **DCIS**				0.067
- DCIS without microinvasion	20 (28.2%) 3 missing values	12 (57.1%)	9 (29.0%)	
- DCIS with microinvasion	3 (4.2%) 3 missing values	1 (4.8%)	0	
- **Grading**				0.658
- G1	7 (10.1%) 5 missing values	3 (14.3%)	4 (13.3%) 1 missing value	
- G2	33 (47.8%) 5 missing values	10 (47.6%)	10 (33.3%) 1 missing value	
- G3	29 (42.0%) 5 missing values	8 (38.1%)	16 (53.3%) 1 missing value	
- **MIB**				0.149
<= 20%	29 (43.9%) 8 missing values	12 (60.0%) 1 missing value	10 (32.3%)	
> 20%	37 (56.1%) 8 missing values	8 (40.0%) 1 missing value	21 (67.7%)	
- **Stadium**				0.102
- Tis N0 M0	7 (10.4%) 7 missing values	4 (21.1%) 2 missing values	9 (39.1%) 8 missing values	
- T1 N0 M0	21 (31.3%) 7 missing values	4 (21.1%) 2 missing values	6 (26.1%) 8 missing values	
- T0/T1 N1 M0	11 (16.4%) 7 missing values	6 (31.6%) 2 missing values	4 (17.4%) 8 missing values	
- T2 N1 M0 - T3 N0, M0	3 (4.5%) 7 missing values	0	2 (8.7%) 8 missing values	
- T0/T1 N2 M0 - T2 N2 M0 - T3 N1/N2 M0	7 (10.4%) 7 missing values	0	0	
- T4 N0/N1/N2 M0	1 (1.5%) 7 missing values	0	0	
- every T N3 M0	1 (1.5%) 7 missing values	1 (5.3%) 2 missing values	0	
- every T every N M1	16 (23.9%) 7 missing values	4 (21.1%) 2 missing values	2 (8.7%) 8 missing values	
- **T-Stage**				0.078
- pT0	1 (1.5%) 6 missing values	0	3 (11.5%) 5 missing values	
- pTis	7 (10.3%) 6 missing values	4 (20.0%) 1 missing value	10 (38.5%) 5 missing values	
- pT1mic	1 (1.5%) 6 missing values	1 (5.0%) 1 missing value	1 (3.8%) 5 missing values	
- pT1a	4 (5.9%) 6 missing values	0	2 (7.7%) 5 missing values	
- pT1b	6 (8.8%) 6 missing values	1 (5.0%) 1 missing value	1 (3.8%) 5 missing values	
- pT1c	20 (29.4%) 6 missing values	6 (30.0%) 1 missing value	5 (19.2%) 5 missing values	
- pT2	21 (30.9%) 6 missing values	6 (30.0%) 1 missing value	4 (15.4%) 5 missing values	
- pT3	7 (10.3%) 6 missing values	2 (10.0%) 1 missing value	0	
- pT4b	1 (1.5%) 6 missing values	0	0	
- **N-Stage**				0.575
- pN0	39 (58.2%) 7 missing values	12 (66.7%) 3 missing values	19 (61.3%)	
- pN1mi	2 (3.0%) 7 missing values	1 (5.6%) 3 missing values	2 (6.5%)	
- pN1a	15 (22.4%) 7 missing values	2 (11.1%) 3 missing values	3 (9.7%)	
- pN1	1 (1.5%) 7 missing values	0	1 (3.2%)	
- pN2a	7 (10.4%) 7 missing values	2 (11.1%) 3 missing values	1 (3.2%)	
- pN3a	3 (4.5%) 7 missing values	1 (5.6%) 3 missing values	0	
- **R**				0.528
- R0	67 (91.8%) 1 missing value	20 (100%) 1 missing value	30 (100%) 1 missing value	
- R1	5 (6.8%) 1 missing value	0	0	
- R1is	1 (1.4%) 1 missing value	0	0	
- **Closest resection margin** (median mm, IQR)	5.0 (2.0;10.0)	5.0 (2.0;7.0)	5.0 (1.0;10.0)	0.823
- **Lymphovascular invasion**				0.407
- L0	25 (56.8%) 30 missing values	6 (54.5%) 10 missing values	14 (73.7%) 12 missing values	
- L1	19 (43.2%) 30 missing values	5 (45.5%) 10 missing values	5 (26.3%) 12 missing values	
- **Extent of disease**				0.298
- Bifocal	5 (11.6%) 31 missing values	1 (11.1%) 12 missing values	1 (5.6%) 13 missing values	
- Multifocal	11 (25.6%) 31 missing values	4 (44.4%) 12 missing values	7 (38.9%) 13 missing values	
- Multicentric	27 (62.8%) 31 missing values	4 (44.4%) 12 missing values	5 (27.8%) 13 missing values	

Breast cancer characteristics, PB: Analysis per breast, BC: breast cancer, RME: radical mastectomy, SSM: skin-sparing mastectomy, NSM: nipple-sparing mastectomy

RFGT was present in 93.9% of breasts following a NSM, 91.2% of breasts after a SSM and 85.2% of breasts following a RME.

The median time between the preoperative and postoperative MRI was 15.7 months (range 12.6; 27.0) in the RME subgroup, 13.5 months (range 12.4; 15.4) in the SSM cohort and 14.0 months (range 11.9; 22.5) in the NSM subgroup (*p* = .093). Details regarding MRI measurements are outlined in Table 4.

**Table 4:** MRI measurements

	RME	SSM	NSM	*p*-VALUE
**Skin and subcutaneous fat tissue**				
- Preoperative				
- Medial (median mm, IQR)	11.5 (8.0; 21.5)	13.0 (10.0; 20.0)	9.5 (7.0; 13.5)	
- Lateral (median mm, IQR)	11.5 (8.0; 15.0)	12.0 (10.0; 15.0)	8.0 (5.5; 11.0)	
- Postoperative				
- Medial (median mm, IQR)	10.0 (5.0; 21.0)	8.0 (4.0; 12.0)	5.0 (3.0; 8.0)	
- Lateral (median mm, IQR)	10.0 (5.0; 24.0)	7.0 (4.0; 11.0)	5.0 (3.0; 7.0)	
**Preoperative fibroglandular tissue (FGT)**				
- Volume (median cm^3^, IQR)	62.37 (27.655; 127.250)	51.59 (35.2; 97.63)	59.10 (36.14; 123.0)	0.732
**Preoperative whole breast volume** (median cm^3^, IQR)	641.75 (373.30; 886.80)	640.50 (475.10; 792.90)	412.75 (244.40; 595.55)	
**Residual fibroglandular tissue (RFGT)**				
- Medial (median mm, IQR)	0 (0;2.0)	0 (0;1.0)	1.0 (0; 2.0)	0.547
- Lateral (median mm, IQR)	1.0 (0;3.0)	1.5 (1.0; 2.5)	2.0 (1.0; 3.0)	0.550
- Retroareolar (median mm, IQR)			3.0 (1.0; 6.0)	
- Volume (median mm^3^, IQR)	100.0 (820.0; 1142.0)	167.0 (20.0; 928.0)	1414.0 (230.0; 3668.0)	< 0.001

MRI measurements. All variables were analysed per breast. RME: radical mastectomy, NSM: nipple-sparing mastectomy, SSM: skin-sparing mastectomy

Regarding the volume of RFGT – statistically higher volumes were present in the NSM-cohort (1414.0 mm^3^ (230.0; 3668.0)) compared to the RME (100.0mm^3^ (820.0; 1142.0)) and SSM-subgroup (167.0mm^3^ (20.0; 928.0)) (*p* < .001).

Furthermore, the following parameters were significantly associated with RFGT volume in univariable analysis: reconstruction (*p* = .012) – with higher RFGT volumes in breasts with immediate primary fixed volume implant reconstruction compared to immediate primary tissue expander, delayed primary tissue expander and no reconstruction; ADM or mesh (*p* = .031) – with higher RFGT volumes in breasts reconstructed with an ADM compared to synthetic mesh and no ADM or synthetic mesh; age (*p* = .022) – with higher RFGT volumes in younger patients; FGT volume (*p* = .012) – with higher RFGT volumes in case of higher FGT volume and whole breast volume (*p* = .030) – with higher RFGT volume in case of higher whole breast volume.

No significant association with RFGT volume in univariable analysis was found for: ME (mastectomy) indication (oncologic vs. prophylactic; *p* = .161), ME volume (*p* = .935), lymph node surgery (sentinel node vs. axillary dissection vs. no lymph node surgery; *p* = .409), surgeon (*p* = .214), BMI (*p* = .507), prior breast operation (breast conserving surgery vs. mastopexy/reduction mammaplasty vs. breast implants vs. no prior breast operation; *p* = .104), neoadjuvant chemotherapy (*p* = .636), prior ME radiotherapy (*p* = .477), post ME radiotherapy (*p* = .848), extent of disease (monofocal vs. bifocal vs. multifocal vs. multicentric; *p* = .809) and T-stage (*p* = .611).

The observed reduction in the postoperative compared to preoperative skin and subcutaneous fat tissue thickness measured medially and laterally reached statistical significance in the NSM cohort (medial 9.5 mm (7.0; 13.5) vs. 5.0 mm (3.0; 8.0), *p* < .001; lateral 8.0 mm (5.5; 11.0) vs. 5.0 mm (3.0; 7.0), *p* = .001) and showed a numerical difference in the RME (medial 11.5 mm (8.0; 21.5) vs. 10.0 mm (5.0; 21.0), *p* = .112; lateral 11.5 mm (8.0; 15.0) vs. 10.0 mm (5.0; 24.0), *p* = .744) and SSM-cohort (medial 13.0 mm (10.0; 20.0) vs. 8.0 mm (4.0; 12.0), *p* = .075; lateral 12.0 mm (10.0; 15.0) vs. 7.0 mm (4.0; 11.0), *p* = .068).

## Discussion

RFGT after a mastectomy can deteriorate the prognosis of a breast cancer patient [7, 10, 16, 17, 21, 22]. Various studies – based on radiologic imaging [1–4] or pathology assessment [5–18] – have demonstrated high prevalence rates of RFGT. The present study aimed to identify risk factors for RFGT, that should be taken into consideration in the course of surgical treatment to potentially prevent or minimize the presence of RFGT.

In the present study higher RFGT volumes were observed following a NSM compared to a SSM and RME (*p* < .001). Notably, existing literature on risk factors of RFGT is contradictory (see Tables 5 and 6). While some studies also identified type of mastectomy as a risk factor for RFGT – with more RFGT being detected following a NSM than a SSM [3, 12, 27] than a total mastectomy [2] – other studies did not (MRI-based evaluation including NSM, SSM and simple mastectomy [20] and pathology-based assessment including total glandular mastectomy and modified radical mastectomy [14]).

**Table 5:** Evaluated risk factors for RFGT I

Procedure and surgeon-related	Association with RFGT		Method	Publication
**ADM or mesh**	Yes		MRI	Deutschmann C, et al.
**Bilateral PME** (vs. unilateral PME and curative ME)	Yes		MRI	Grinstein O, et al. Surg Oncol. 2019 [1]
**Breast reconstruction**	Yes	with an implant/tissue expander	MRI	Deutschmann C, et al.
		with a flap	MRI	Giannotti DG, et al. Int J Radiat Oncol Biol Phys. 2018 [2]
**Date of surgery** (more RFGT with a more recent date of surgery)	Yes		MRI	Zippel D, et al. Clin Imaging. 2015 [3]
**Indication of surgery**	Yes	prophylactic> therapeutic	MRI	Giannotti DG, et al. Int J Radiat Oncol Biol Phys. 2018 [2]
	No		MRI	Woitek R, et al. Eur J Radiol. 2018 [4]
				Zippel D, et al. Clin Imaging. 2015 [3]
				Deutschmann C, et al.
			Pathology	Papassotiropoulos B, et al. Ann Surg Oncol. 2019 [5]
**Lymph node surgery**	No		MRI	Deutschmann C, et al.
**Mastectomy volume**	No		MRI	Deutschmann C, et al.
**Skin flaps** (> 5 mm)	Yes		MRI and US	Andersson MN, et al. J PlastReconstr Aesthet Surg. 2022 [6]
			MRI	Grinstein O, et al. Surg Oncol. 2019 [1]
				Baltzer HL, et al. Ann Surg Oncol. 2014 [7]
				Zippel D, et al. Clin Imaging. 2015 [3]
				Giannotti DG, et al. Int J Radiat Oncol Biol Phys. 2018 [2]
				Dietzel F, et al. Cancers (Basel). 2023 [8]
			Pathology	Torresan RZ, et al. Ann Surg Oncol. 2005 [9]
				Cao D, et al. Ann Surg Oncol. 2008 [10]
	No		MRI	Woitek R, et al. Eur J Radiol. 2018 [4]
**Surgeon**	Yes		MRI	Dietzel F, et al. Cancers (Basel). 2023 [8]
			Pathology	Papassotiropoulos B, et al. Ann Surg Oncol. 2019 [5]
	No		MRI	Woitek R, et al. Eur J Radiol. 2018 [4]
				Deutschmann C, et al.
			Pathology	Dreadin J, et al. Breast J. 2012 [11]
**Surgical units with lower caseload**	Yes		MRI	Grinstein O, et al. Surg Oncol. 2019 [1]
**Type of incision**	No		Pathology	Papassotiropoulos B, et al. Ann Surg Oncol. 2019 [5]
**Type of mastectomy**	Yes	NSM> SSM	MRI	Woitek R, et al. Eur J Radiol. 2018 [4]
				Dietzel F, et al. Cancers (Basel). 2023 [8]
			Pathology	Papassotiropoulos B, et al. Ann Surg Oncol. 2019 [5]
		NSM> SSM> total mastectomy	MRI	Giannotti DG, et al. Int J Radiat Oncol Biol Phys. 2018 [2]
				Deutschmann C, et al.
	No	Including NSM, SSM and simple mastectomy	MRI	Skoglund MA, et al. Acta Radiol. 2023 [12]
		Including total glandular mastectomy vs. modified radical mastectomy	Pathology	Barton FE, et al. Plast Reconstr Surg. 1991 [13]

Evaluated risk factors for RFGT I (in alphabetical order), RFGT: residual fibroglandular breast tissue, ADM: acellular dermal matrix, PME: prophylactic mastectomy, ME: mastectomy, US: ultrasound

**Table 6:** Evaluated risk factors for RFGT II

Patient-related	Association with RFGT		Method	Publication
**Age**	Yes		MRI	Zippel D, et al. Clin Imaging. 2015 [1]
				Deutschmann C, et al.
	No		Pathology	Papassotiropoulos B, et al. Ann Surg Oncol. 2019 [2]
				Dreadin J, et al. Breast J. 2012 [3]
				Torresan RZ, et al. Ann Surg Oncol. 2005 [4]
**BMI**	No		Pathology	Papassotiropoulos B, et al. Ann Surg Oncol. 2019 [2]
				Dreadin J, et al. Breast J. 2012 [3]
				Torresan RZ, et al. Ann Surg Oncol. 2005 [4]
**Breast density**	Yes		MRI	Dietzel F, et al. Cancers (Basel). 2023 [5]
	No		Pathology	Torresan RZ, et al. Ann Surg Oncol. 2005 [4]
**Breast volume**	Yes	FGT volume	MRI	Deutschmann C, et al.
		Whole breast volume	MRI	Deutschmann C, et al.
		Breast volume	MRI	Dietzel F, et al. Cancers (Basel). 2023 [5]
	No		Pathology	Papassotiropoulos B, et al. Ann Surg Oncol. 2019 [2]
				Torresan RZ, et al. Ann Surg Oncol. 2005 [4]
**Clinical and pathological staging**	No		Pathology	Torresan RZ, et al. Ann Surg Oncol. 2005 [4]
**Distance < 1 mm between specimen surface and specimen breast tissue**	Yes		Pathology	Papassotiropoulos B, et al. Ann Surg Oncol. 2019 [2]
**Estrogen receptor status**	No		Pathology	Dreadin J, et al. Breast J. 2012 [3]
**Extend of disease**	No		MRI	Deutschmann C, et al.
**Menopausal status**	No		Pathology	Torresan RZ, et al. Ann Surg Oncol. 2005 [4]
**Neoadjuvant chemotherapy**	No		MRI	Deutschmann C, et al.
			Pathology	Torresan RZ, et al. Ann Surg Oncol. 2005 [4]
**Parity**	No		Pathology	Torresan RZ, et al. Ann Surg Oncol. 2005 [4]
**Patient height**	Yes		MRI	Giannotti DG, et al. Int J Radiat Oncol Biol Phys. 2018 [6]
**Presence of an extensive in situ component**	No		Pathology	Torresan RZ, et al. Ann Surg Oncol. 2005 [4]
**Prior breast operation**	No		MRI	Deutschmann C, et al.
**Radiotherapy**	No	Prior to mastectomy	MRI	Deutschmann C, et al.
		Post mastectomy	MRI	Deutschmann C, et al.
**Tumor size**	No		MRI	Deutschmann C, et al.
			Pathology	Dreadin J, et al. Breast J. 2012 [3]

Evaluated risk factors for RFGT II (in alphabetical order), RFGT…residual fibroglandular breast tissue

Woitek R et al. [3] ascribed the higher amount of RFGT following a NSM compared to a SSM to the more linear incision in NSM and hence limited surgical accessibility in comparison to the circular incision around the areola in SSM.

Furthermore, the higher volume of RFGT following a NSM compared to other types of mastectomy can be explained by the preservation of the skin envelope and the NAC. Owing to the frequent irregularity or lack of the superficial fascia of the breast – which is considered as landmark of dissection [18, 28] – the identification of the correct anatomic dissection plane in the course of skin flap preparation is often impeded and results in incomplete FGT removal. Furthermore, the thickness of the subcutaneous fat tissue was shown to be inhomogeneous even within one breast [12] complicating tissue preparation and attributing to the higher likelihood of RFGT when the skin envelope is preserved.

Additionally, terminal duct lobular unit (TDLU) density was found to be higher in the NAC than in the adjacent skin [12] explaining the higher risk of RFGT if the NAC is preserved. High prevalence rates and RFGT location predominantly in the retroareolar area were reported by various authors [4, 6, 12, 20, 29, 30].

Notably, despite the higher likelihood of RFGT following NSM compared to any other mastectomy type, NSM in comparison to SSM or total mastectomy has shown no significant difference in recurrence rates or overall survival [31–34]. This highlights the significance of tumor biology rather than surgical techniques regarding the oncologic outcome of breast cancer patients.

In the present study RFGT volume was significantly associated with reconstruction (*p* = .012) – with higher RFGT volumes in breasts with immediate primary fixed volume implant reconstruction compared to immediate primary tissue expander reconstruction, delayed primary tissue expander reconstruction and no reconstruction, as well as ADM or mesh (*p* = .031) – with higher RFGT volumes in breasts reconstructed with an ADM compared to reconstruction with a synthetic mesh or no use of an ADM or synthetic mesh.

This might be explained by the aspiration to preserve a thicker skin envelope, which has been associated with RFGT in literature [1, 2, 4, 10, 17, 23, 24, 27], if implant-based reconstruction is performed in order to ensure flap perfusion and viability. Notably, Roy De Vita et al. [35] showed a statistically significant association between complications after NSMs and skin flaps of less than 5 mm. Frey JD et al. [36] identified a NSM flap thickness of less than 8.0 mm to be an independent predictor of ischemic complications.

Further procedure and surgeon-related risk factors for RFGT analysed in literature are displayed in Table 5.

Regarding patient related risk factors for RFGT, RFGT volume was significantly associated with patient age in the present study (*p* = .022) – with higher RFGT volumes found in younger patients. This might be explained by the high breast density in young patients (which has been associated with RFGT in literature [27]). Yet, contrary to the present study, Zippel D, et al. [1] detected more RFGT in older patients. Other authors found no association of RFGT with age at all [10–12].

RFGT volume was also associated with preoperative FGT volume in the present study (*p* = .012) – with higher RFGT volumes in case of higher FGT volumes – and preoperative whole breast volume (*p* = .030) – with higher RFGT volume in case of higher whole breast volume. Similar results were shown by Dietzel F, et al. [27]. This might be explained by the technically more difficult tissue preparation in larger breasts.

Further patient-related risk factors for RFGT studied in literature are displayed in Table 6.

Notably, contradictory findings of risk factors of RFGT in literature including the present study might be explained by methodological differences between the studies: MRI vs. pathology-based RFGT assessment and different RFGT sampling techniques in pathology -based studies (sample collection from the superficial dissection plane of the mastectomy specimen versus mastectomy cavity). In addition, the relatively small sample cohorts studied and the often retrospective study design also contributed to the heterogeneity of the results.

We furthermore, evaluated the tissue envelope thickness of the analysed breasts and found a reduction in the postoperative compared to preoperative skin and subcutaneous fat tissue thickness reaching statistical significance in the NSM cohort (medial *p* < .001, lateral *p* = .001) and showing a numerical difference in the RME and SSM-cohort. Notably, as skin flaps of less than 5 mm have been associated with ischemic complications [35], we suggest preoperative planning and determination of the patients’ individual target flap thickness – in accordance with Woitek R et al. [3] – in order to ensure thorough preservation of the skin and subcutaneous fat tissue thickness as well as complete removal of FGT.

Limitations of the present study include the retrospective study design and the partially missing data as well as the relatively small sample cohort.

## Conclusions

In conclusion, identification of risk factors for RFGT is a first step to potentially prevent the remaining of FGT after mastectomy in the future. The described reduction in the post- compared to preoperative skin and subcutaneous fat tissue thickness should be avoided considering the known associated increase in risk for ischemic complications.

### Electronic supplementary material

Below is the link to the electronic supplementary material.

Supplementary Material 1

## Data Availability

The datasets generated and analysed during the current study are not publicly available due to ongoing data analysis and further manuscript preparation on additional aspects of the topic but are available from the corresponding author on reasonable request.

## Abbreviations

ADM: Acellular dermal matrix
FGT: Fibroglandular tissue
ME: Mastectomy
MRI: Magnetic resonance imaging
NAC: Nipple areola complex
NSM: Nipple-sparing mastectomy
RFGT: Residual fibroglandular breast tissue
RME: Radical mastectomy
SSM: Skin-sparing mastectomy
TDLU: Terminal duct lobular unit
