# Supplementary material for: Risk factors for residual fibroglandular breast tissue following a mastectomy - an overview and retrospective cohort study
Source: BMC Cancer. 2024 Jul 18;24:856. doi: 10.1186/s12885-024-12491-4 (PMC11256640; doi:10.1186/s12885-024-12491-4)
Supplement: Supplementary file 1 — Supplementary Material 1 [file 12885_2024_12491_MOESM1_ESM.docx]

| **TABLE S1: Reason for study exclusion**  Multiple answers per patient possible | **Number of patients/breasts** |
| --- | --- |
| **Post-ME MRT not available** | 510/594 |
| **Autologous breast reconstruction** (including flap reconstruction and lipofilling) | 52/56 |
| **Clinical data not available** | 31/36 |
| **Second-look resection** | 28/30 |

Table S1: Reasons for study exclusion, ME…mastectomy
